# Can Empathy Help Individuals and Society? Through the Lens of Volunteering and Mental Health

**DOI:** 10.3390/healthcare9111406

**Published:** 2021-10-20

**Authors:** Yang Woon Chung, Seunghee Im, Jung Eun Kim

**Affiliations:** 1 College of Economics and Business Administration, University of Suwon, Hwaseong-si 18323, Korea; jywchung@suwon.ac.kr; 2 Department of Child and Family Welfare, University of Suwon, Hwaseong-si 18323, Korea; jekim@suwon.ac.kr

**Keywords:** mental health, volunteering, cognitive empathy, affective empathy, gender

## Abstract

(1) Background: Empathy affects an individual’s decision to participate in volunteering, and volunteering, in turn, influences mental health. Intriguingly, studies have been limited in exploring underlying mechanisms and boundary conditions for the relationship between empathy and mental health. Furthermore, volunteering studies have overlooked the multi-dimensionality of empathy. Therefore, this study seeks to contribute to extant literature by investigating the mediating effect of volunteering for the relationships between cognitive and affective empathy and mental health and the moderating effect of gender for the relationship between empathy and volunteering. (2) Methods; Data were collected using a survey in South Korea and consisted of 301 full-time employees who voluntarily engaged in their corporate volunteer programs. Furthermore, they voluntarily participated in the study. The hypotheses were tested with path analysis and a group comparison was also conducted. (3) Results: Volunteering was found to mediate the relationships between cognitive empathy and affective empathy with mental health. In addition, gender moderated the relationship between empathy and volunteering. (4) Conclusions: As the study found empathy to increase individuals’ engaging in volunteering activities which then improved mental health, the study supports extant theoretical frameworks on empathy and volunteering. Moreover, the study found gender differences on empathy and volunteering; thereby supporting and contributing to extant literature.

## 1. Introduction

Volunteering has become an important aspect in our lives in that it provides benefits to individuals, organizations, communities, and societies [1]. In particular, the positive effects on volunteers have been well documented, showing that volunteering is related to physical, cognitive, and psychosocial outcomes such as increased levels of mental health, physical health, social interactions, and prosocial attitudes and behaviors e.g., [2]. The most frequently investigated outcome of volunteering is mental health [3,4] as studies have found volunteering to increase psychological well-being, life satisfaction, and happiness while reducing depression, psychological distress, stress, and burnout e.g., [5]. Moreover, volunteering has become an important facet within organizations because employee volunteering has been found to have numerous positive effects on employees. For instance, previous studies have demonstrated that when employees participate in volunteering activities, it results in higher levels of positive emotions, self-esteem, happiness, life satisfaction, psychological well-being, and lower depression levels [6,7]. 

According to the conceptual framework on employee volunteering of Rodell et al. [6], individual factors such as personality traits can influence an individual’s decision to participate in volunteering. They argued that personality traits can affect mental health through employee volunteering and that underlying mechanisms have been overlooked within literature. Among the personality traits within literature, empathy has been found to be the most essential factor that stimulates volunteering [8] due to an individual’s altruistic motives [9] and generosity [10]. Moreover, empathy has been suggested to be multi-dimensional in nature. Cognitive empathy refers to the ability to understand another individual’s feelings [11] and affective empathy refers to being concerned with emotional experiences that are caused by emotional stimuli [12]. Although there are arguments against the separation of the two concepts e.g., [13,14], the two concepts can be separated but have close interactions [15] as Strayer [16] argued that affective empathy is the content of empathy while cognitive empathy is the process where the content is established. However, many of the prior studies have considered empathy as a single dimension [17], or have focused on only one particular dimension of empathy e.g., [18]. 

It is notable that there has been a recent attempt to consider the multi-dimensionality of empathy when examining the relationships between volunteering and empathy. For example, Kim and Kou [19] differentiated the dimensions of empathy and found that only affective empathy among the components of empathy had a positive relationship with volunteering. Despite these efforts to reflect the multi-dimensionality of empathy, less attention has been given to the different effects of each dimension of empathy on volunteering. 

In addition, previous studies have found that there can be gender differences for the relationships between empathy and prosocial behavior [20]. Although recent studies have suggested that gender can moderate the relationship between empathy and volunteering [21], there is a lack of empirical research that has examined the moderating effect of gender considering the multi-dimensionality of empathy and its outcomes.

Therefore, this study seeks to contribute to extant literature by investigating the mediating effect of volunteering for the relationships between cognitive and affective empathy and mental health and the moderating effect of gender for the relationship between empathy and volunteering. Thus, the study considers the multi-dimensionality of empathy when investigating the relationships between empathy, volunteering, and mental health and examines the different effects of cognitive and emotional empathy on volunteering by gender. 

## 2. Hypothesis Development 

Empathy has generally been characterized as the tendency to be sensitive to others and to vicariously experience the emotions, feelings, and thoughts of others c.f., [22]. Empathy has been conceptualized as a combination of inter-related aspects of emotion recognition in oneself and others, affective responsiveness by sharing the emotional experiences of others, and perspective taking by cognitively perceiving the perspectives of others [23]. Empathy allows individuals to share and understand one another’s feelings and intentions [24]. Empathy involves the experience of emphatic emotions that generate interest and care for the welfare of others where empathy is felt [25]. Hence, emphatic emotions toward a person leads to helping that person because empathy involves an individual imagining another person’s perspective and feeling care and concern for the person [26].

Empathy has been suggested to lead to behavioral outcomes e.g., [27]. For instance, scholars have argued that empathy is associated with prosocial behavior e.g., [28,29,30]. Penner [31] suggested that personality traits such as proactive personality are related to volunteering. His prosocial personality model proposed two personality factors: other-oriented empathy and helpfulness. Other-related empathy refers to prosocial thoughts and feelings while helpfulness refers to physical actions. He argued that other-oriented empathy is an important antecedent to volunteering because it includes prosocial feelings and thoughts and the tendency to perceive empathy, concern, and responsibility for the welfare of others. In addition, other-oriented empathy includes empathic concern, perspective taking, social responsibility, other-oriented moral reasoning, and mutually concerned moral reasoning [31]. Further, research has shown that other-oriented empathy differentiates individuals who volunteer and do not volunteer, the amount of time spent on volunteering, and the intentions to volunteer [31,32,33]. Hence, studies have found other-oriented empathy to be associated with volunteering e.g., [31,34,35].

Due to the nature of empathy, empathy can naturally result in positive outcomes. In general, empathy can have a positive effect on mental health due to its prosocial nature. For example, empathy has been suggested to enhance well-being because when individuals are empathetic to others, not only will they perceive positive feelings like happiness because they are doing something good for others, but others will feel grateful toward them which can positively influence individuals. Moreover, empathy allows individuals to feel connected with others which then can result in increased happiness and positive affect [36]. Hence, studies have found empathy to be positively associated with life satisfaction, well-being, and self-efficacy e.g., [36,37,38,39].

Furthermore, research has suggested that there are numerous positive benefits from volunteering such as need satisfaction, morale, identification, and mental health. Volunteering and mental health have been frequently studied and research has long argued that volunteering results in positive mental health because when individuals volunteer, psychological resources are increased and that helping others can boost one’s self-esteem and positive mood states e.g., [40,41]. As studies have found volunteering to improve mental health e.g., [42,43], research has also found volunteering to lessen depression symptoms e.g., [44] and stress e.g., [45]; thus, it can be natural to posit that volunteering can mediate the relationship between empathy and mental health.

**Hypothesis** **1.** *Volunteering will mediate the relationship between cognitive empathy and mental health.*

**Hypothesis** **2.** *Volunteering will mediate the relationship between affective empathy and mental health.*

Gender socialization theory is a pivotal theoretical framework to explain gender differences [46]. This theory assumes gender specific socialization experiences. As a result, males are socialized towards masculine behaviors, including instrumentality, assertion, and competitiveness, whereas females are socialized towards nurturing, expressive, and caring behaviors [47]. Parallel with this theory, some studies have argued gender-specific socialization may affect its relationships with affective empathy and cognitive empathy [48]. For instance, Eisenberg et al. [20] mentioned that cognitive empathy plays a more important role in prosocial behavior for males, while affective empathy may be a stronger facilitator to show prosocial behavior for females. Similarly, Longobardi et al. [49] suggested the moderating effect of gender in the association between empathy and prosocial behavior, demonstrating that cognitive empathy has a positive effect on prosocial behavior in males but not in females. 

In addition, volunteering literature found volunteering to be linked to different values across genders [8]. Females are more likely to associate volunteering with an expression of caring while males are more likely to perceive volunteering as the accomplishment of specific tasks [50]. Accordingly, cognitive processes for males and emotional processes for females may be important motives for engaging in volunteer activities. In this regard, this study proposes that gender will moderate the relationship between empathy and volunteering. Specifically, we expect that the effect of cognitive empathy on volunteering will be greater in males than in females, as males tend to be more motivated by cognitive motives. In a similar manner, we predict that the influence of affective empathy on volunteering will be stronger in females than in males since females have a tendency to be facilitated by affective motives. Therefore, we propose the following:

**Hypothesis** **3.** *Gender will moderate the relationship between empathy and volunteering as there will be gender differences in the effect of empathy on volunteering.*

## 3. Materials and Methods 

### 3.1. Sample and Data Collection

Full-time employees from numerous organizations in South Korea voluntarily participated in this study. The organizations that were selected operated various employee volunteering programs such as assisting volunteer organizations with mentoring, helping, and educating children in need. Self-reported surveys were used to collect data and in order to control for common method bias, the purpose of the study and protection of anonymity of the respondents were stated on the cover of the questionnaire [51]. Data were collected from January to February 2019. 

The questionnaires were distributed to 400 employees and 306 questionnaires were returned (76.5% response rate). Out of the 306 questionnaires, 301 were usable after excluding cases with missing values. A preliminary analysis revealed that the average age of respondents was 34.1 years old (S.D. = 8.21) and average tenure was 9.1 years (S.D. = 7.51). 54% of the respondents were male and 67.6% had a college degree or higher.

### 3.2. Measures

The measures selected in the study were originally developed in English and all of the measures were translated into Korean. The back-translation method was used to ensure the quality of the translations [52]. The questionnaire used a 7-point Likert scale from 1, “strongly disagree,” to 7, “strongly agree”, for all measures.

Cognitive empathy was measured with Chowdhury and Fernando’s [53] four-item scale. Sample items are: “I believe that there are two sides to every question and try to look at them both,” and “When I’m upset at someone, I usually try to ‘‘put myself in his or her shoes” for a while.”

Affective empathy was measured with Chowdhury and Fernando’s [53] four-item scale. Sample items are: “I often have tender, concerned feelings for people less fortunate than me,” and “Other people’s misfortunes do not usually disturb me a great deal(R).”

Volunteering was measured with Rodell et al.’s [6] three-item sale. Sample items are: “I devote my energy toward a volunteer group,” and “I engage in activities to support a volunteer group.”

Mental health was measured with Hays et al.’s [54] five-item scale. Sample items are: “In general, my mental health, including the mood and ability to think, is good,”, “I am often bothered by emotional problems.”

Several sociodemographic variables such as gender and age were measured. Gender was measured to be used as a moderating variable. Age was measured to control potential inference effects in the analyses. 

## 4. Results

Descriptive statistics summarizing the characteristics of the dataset are provided in Table 1 with the results from the correlation analysis. 

Before testing the hypotheses, confirmatory factor analysis (CFA) was performed, and the distinctiveness of cognitive and affective empathy was confirmed as shown in Table 2. 

To test the hypotheses, path analysis was conducted using Mplus 8.0 and a group comparison by gender was performed. A group analysis was employed to find the differences across gender [8,48] and nested models were compared step by step using a chi-square difference test following previous research [55,56]. The model was fitted using the maximum likelihood mean-variance adjusted (MLMV) estimator since it allows the DIFFTEST command for the chi-square difference test and is known to yield a better estimation [57,58]. Constraints were released based on the literature reviewed in the previous section e.g., [8], which means the paths from cognitive and affective empathy were freed. The models tested were (1) a model with no constraints (M1), (2) a model with a path from volunteering to mental health being constrained (M2), (3) a model with paths from cognitive and affective empathy to mental health as well as the path from volunteering to mental health being constrained (M3), and (4) a model with all paths constrained to be equal across gender (M4).

Model fit indices were reported and the cutoff criteria were determined based on previous research [59]. A model is considerd to have a good fit with a root mean square error pf approximation (RMSEA) being less than 0.06, a comparative fit index (CFI) and Tucker–Lewis index (TLI) being above 0.95. 

Results from the chi-square difference tests between the nested models showed that M1 did not significantly improve the model fit compared to M2 (χ ^2^ = 2.55, *p* = 0.11) and M3 was preferred compared to M2 (χ ^2^ = 1.90, *p* = 0.39). M3 did not significantly improve the model fit compared to M4, while the fit indices of M3 showed a better fit. More detailed results for M4 (a model with cross-group equality constraint for all paths) and M3 (a model with 2 path released—from cognitive and affective empathy to volunteering) are described below.

**Figure 1:**
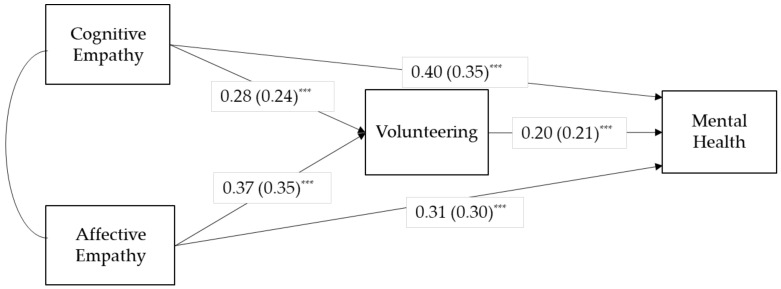
Results from the constrained model. Note: Standardized path coefficients are presented in parentheses. *** *p* < 0.001.

Table 3 and Figure 1 show the results from the path analysis of M4, a constrained model. The mediating role of volunteer participation was found to be significant. To test the significance of mediation, both the delta method and bootstrapping were conducted [59]. Results of the delta method are presented in Table 3 and the bias-corrected bootstrapping showed that 95% confidence intervals for all indirect paths did not include zero. Confidence intervals for the indirect efffect was (0.02, 0.10) for cognitive empathy and (0.03, 0.13) for affective empathy, respectively. Thus, the results supported Hypotheses 1 and 2.

**Figure 2:**
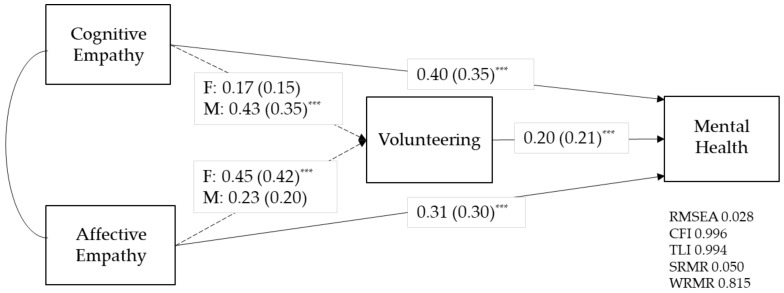
Results from group analysis by gender. Note: Standardized path coefficients are presented in parentheses. Dash lines exhibit the paths showing gender differences. *** *p* < 0.001.

Estimates from M3, a model with 2 released paths (from cognitive empathy to volunteering, and affective empathy to volunteering, respectively) are illustrated in Table 4 and Figure 2. 

Hypothesis 3 was supported as gender differences were found in the two paths. For males, the path from cognitive empathy to volunteering was significant (B = 0.43, β **=** 0.35, *p* = 0.001) and the path from affective empathy to volunteering was not significant (B = 0.23, β = 0.20, *p* = 0.083). For females, the effect of cognitive empathy on volunteering was not significant (B = 0.17, β = 0.15, *p* = 0.094) while affective empathy showed a significant effect (B = 0.45, β = 0.42, *p* < 0.001).

As mentioned previously, while both M4 with all paths constrained to be equal across gender and M3 with two released paths fitted the data very well, the less restricted model M3 showed a better fit (RMSEA = 0.028, CFI = 0.996, TLI = 0.994) compared to the constrained model (RMSEA = 0.031, CFI = 0.994, TLI = 0.993). We performed a chi-square difference test and the result was not significant (χ ^2^ = 2.41, *p* = 0.30), which means we cannot reject the null hypothesis that the less restricted model does not significantly improve the model fit. 

## 5. Discussion

According to the conceptualized framework of Rodell et al. [6], the study found individual differences can significantly impact individuals in engaging in volunteering activities. Cognitive and affective empathy were found to be positively related to employee volunteering. Since empathy focuses on an individual’s perspective taking and empathic concern and has been often associated with prosocial behavior [29,60], empathy can allow individuals to engage in volunteering activities. Furthermore, the study results can be strengthened due to the study being conducted in Korea. Collectivistic cultures tend to focus on the collective as a whole, engage in social behaviors that are significantly influenced by norms, duties, and obligations, and emphasize the importance of relationships [61,62]. In this regard, collectivism has been found to be associated with empathy e.g., [63], thus further supporting the study findings.

Volunteering was found to improve mental health. Volunteering not only offers a valuable service to society but also can result in positive individual outcomes such as improved mental health for those that engage in volunteering activities. The findings of the study were consistent with extant research e.g., [64,65] as volunteering was found to be positively related to psychological well-being. Subsequently, volunteering was found to mediate the relationship between cognitive empathy and affective empathy with mental health; thereby coinciding with the employee volunteering framework of Rodell et al. [6].

Another finding for this study was that gender moderated the relationship between empathy and volunteering, and as hypothesized, gender differences were found. For males, the effect of cognitive empathy on volunteering was significant, while the effect of affective empathy on volunteering was not significant. On the other hand, in females, the effect of cognitive empathy on volunteering was not significant, whereas affective empathy showed a significant effect on volunteering. This finding supports gender socialization theory as it helps explain gender differences in social behavior in order to fulfill his/her needs formed through gender specific socialization experiences [46]. The results of the study is also consistent with prior research which suggested that there are gender differences in the value of promoting volunteering engagement [8]. As males perceive volunteering as a task-oriented social behavior, cognitive processes such as cognitive empathy tend to facilitate volunteering. In contrast, because females perceive volunteering as a social behavior of caring and emotional processes, females can be more likely to engage in volunteering activities. 

### 5.1. Implications

The study proposed that volunteering is a proximal outcome of cognitive and affective empathy that can link the distal relationship between empathy and mental health. In this notion, the study is in alignment with the conceptual framework of Rodell et al. [6] as personality traits such as empathy can influence volunteering which then results in personal outcomes such as mental health. Moreover, our findings add to literature regarding the relationships between empathy, volunteering, and mental health by incorporating the moderating role of gender. Since prior studies have mainly examined the direct effect of empathy to explain how empathy influences volunteering e.g., [31], there has been a dearth of studies on the boundary conditions for the relationship between empathy and volunteering. Furthermore, the study is valuable in that, beyond the direct effect of empathy on volunteering, it helps elucidate the interaction effect of empathy with gender on volunteering. Last, the study suggests that it is important to consider the multi-dimensionality of empathy by demonstrating that there are gender differences in the influence of cognitive and emotional empathy on volunteering.

Studies have long argued that positive workplace attitudes lead to higher performance e.g., [66]. Similarly, Quick [67] argued that when healthy work exists, employees feel good which then results in high performance levels and well-being. In this light, organizations and managers should consider increasing the awareness of employee volunteer programs as it can result in positive individual outcomes such as increased mental health which then can have a positive impact on individual and organizational performance. Furthermore, it is important for organizations to understand why people volunteer. Findings of the study suggested that cognitive empathy for males and affective empathy for females are significant motivators in engaging in volunteering activities. A better understanding of gender differences in volunteering with empathy can enable organizations to make better decisions to facilitate volunteering participation. Therefore, organizations can attempt to identify demographic characteristics (i.e., gender) to select segments at which appeals can be directed, and tailor their message more effectively according to the segments. For example, the rational appeal for the men and the emotional appeal for the females can be effectively used in order to encourage volunteering. 

### 5.2. Limitations and Future Directions

Despite the valuable findings of the study, the current study has a few limitations. First, our research design may have weaknesses in inferences of the causal relationships. The study employed a cross-sectional design and all of the measures were simultaneously measured. In addition, respondents were self-selected into participating in the study; thus, selection bias can be of concern. Future studies should take steps to further validate the causal relationships by applying more rigorous approaches such as longitudinal designs or experiments. Second, this study did not take into account any curvilinear relationships between the study variables. For example, studies have suggested that the relationships between volunteering and health outcomes can be curvilinear [68,69]. Future studies need to consider the curvilinear relationships between the variables in order to deepen understanding and knowledge related to employee volunteering. Third, the study was conducted in Korea, which focuses on collectivism. According to Hofstede’s cultural dimensions theory, national cultures influence the values of its members and these values are related to their behavior [70]. In this regard, volunteering research has identified that volunteering is related to cultural differences in the value of prosociality [3]. Although being prosocial is valued in most cultures, the degree of being prosocial varies from culture to culture [71]. Furthermore, gender differences have been examined in volunteering motivation and behavior i.e., [72]. However, gender differences in volunteering have been mainly examined in individualistic cultures, such as the United States i.e., [72] and European nations i.e., [73]. Therefore, future studies need to replicate this study in other cultures or conduct cross-cultural studies to deepen our knowledge. Last but not least, researchers need to consider the possible of social desirability bias, which is known to be more prevalent in collectivistic societies [74], and the methods to reduce it. Podsakoff, MacKenzie, Lee and Lee [75] have suggested several methods to reduce common method biases such as collecting data from different sources such as peers and supervisors, temporal separation of measurement, and conducting a Harman’s single-factor test. In addition, scales such as the Marlowe–Crowne social desirability scale can be utilized to identify the presence of social desirability bias in the respondents’ responses [76]. 

## 6. Conclusions

Employees with empathic concern can gain numerous individuals, organizational, and societal benefits. As the study found empathy to increase employees engaging in volunteering activities which then increased individual mental health, it supports extant theoretical frameworks on empathy and volunteering. Moreover, the study found gender differences on empathy and volunteering, thereby contributing to extant literature.

## Figures and Tables

**Table 1:** Means, standard deviations, and zero-order correlations (*n* = 301).

	Mean	SD	1	2	3	4	5	6	7
1. Gender (male = 1)	0.53	0.50							
2. Age	34.14	8.21	0.43 ***						
3. Cognitive empathy	5.43	0.87	0.13 *	0.15 **	0.18 **	(0.90)			
Male	5.54	0.85							
Female	5.31	0.88							
4. Affective empathy	5.47	0.92	0.10	0.14 *	0.18 **	0.67 ***	(0.92)		
Male	5.55	0.88							
Female	5.37	0.96							
5. Volunteering	5.22	1.03	−0.02	0.05	0.03	0.45 ***	0.48 ***	(0.92)	
Male	5.20	1.04							
Female	5.24	1.03							
6. Mental health	5.50	1.01	0.10	0.13 *	0.17 **	0.64 ***	0.62 ***	0.50 ***	(0.94)
Male	5.60	0.99							
Female	5.39	1.02							

Note: Reliabilities are in parentheses (Cronbach alpha coefficient). * *p* < 0.05, ** *p* < 0.01, *** *p* < 0.001.

**Table 2:** Confirmatory factor analysis (CFA) results for the empathy variables.

	χ^2^	df	CFI	TLI	RMSEA
1-factor (empathy)	519.01	101	0.91	0.89	0.12
2-factor (cognitive and affective empathy)	210.17	98	0.98	0.97	0.06

Note: df = degree of freedom; CFI = Comparative Fit Index; TLI = Tucker-Lewis Index; RMSEA = Root Mean Square Error of Approximation.

**Table 3:** Coefficients from the constrained model.

From	To	Direct Effect			Indirect Effect			Total Effect		
		B	β	SE	B	β	SE	B	β	SE
Cognitive empathy	Volunteering	0.28 ***	0.24	0.08	-	-	-	0.28 ***	0.24	0.08
	Mental health	0.40 ***	0.35	0.07	0.06 **	0.05	0.02	0.46 ***	0.40	0.07
Affective empathy	Volunteering	0.37 ***	0.35	0.08	-	-	-	0.37 ***	0.35	0.08
	Mental health	0.31 ***	0.30	0.07	0.07 **	0.07	0.03	0.39 ***	0.37	0.07
Volunteering	Mental health	0.20 ***	0.21 ***	0.05	-	-	-	0.20 ***	0.21 ***	0.05

Note: ** *p* < 0.01, *** *p* < 0.001. Age was controlled, while the coefficient was not significant (results with age available upon request).

**Table 4:** Coefficients from group analysis by gender.

From	To	Female			Male		
		B	β	SE	B	β	SE
Cognitive empathy	Volunteering	0.17	0.15	0.10	0.43 ***	0.35	0.13
	Mental health	0.40 ***	0.35	0.07	0.40 ***	0.34	0.07
Affective empathy	Volunteering	0.45 ***	0.42	0.09	0.23	0.20	0.14
	Mental health	0.31 ***	0.30	0.07	0.31 ***	0.28	0.07
Volunteering	Mental health	0.20 ***	0.21	0.05	0.20 ***	0.21	0.05

Note: *** *p* < 0.001. Age was controlled, while the coefficient was not significant (results with age available upon request).

## Data Availability

The data presented in this study are available from the corresponding author upon reasonable request.
